# Del-Nido cardioplegia in cardiac surgery for elderly patients: a propensity score-matched analysis

**DOI:** 10.1186/s13019-023-02269-3

**Published:** 2023-04-25

**Authors:** Wenda Gu, Hongkun Qing, Xiang Luo, Xin Zang, Kan Zhou, Haijiang Guo, Chengbin Zhou, Huiming Guo, Jian Liu

**Affiliations:** Department of Cardiovascular Surgery, Guangdong Cardiovascular Institute, Guangdong Provincial People’s Hospital (Guangdong Academy of Medical Sciences), Southern Medical University, 106 Zhongshan Er Road, Guangzhou, 510080 China

**Keywords:** cardioplegia, Del-Nido, Elderly cardiac surgery, Myocardial protection, Cardiopulmonary bypass

## Abstract

**Objectives:**

To compare the safety and efficacy of del-Nido cardioplegia (DNC) with traditional 4:1 cold blood cardioplegia (CBC) in coronary artery bypass grafting and/or valve surgeries in elderly patients.

**Methods:**

The present study is a retrospective case-series study that included 302 consecutive patients aged 70 years and over who underwent on-pump valve surgery and/or coronary artery bypass graft (CABG). DNC was administered to 90 patients and CBC to 212 patients. After propensity-score matching, 89 pairs were compared. The safety and efficacy were analyzed between the two groups.

**Results:**

The DNC group had a similar mortality (3.4% vs. 5.6%, OR = 0.79, *P* = 0.720) and extracorporeal membrane oxygenation (ECMO) implantation rate (1.1% vs. 2.2%, OR = 0.75, *P* = 1.000) to the CBC group, a lower incidence of postoperative intra-aortic balloon pump (IABP) implantation (1.1% vs. 9.0%, OR = 0.54, *P* = 0.034) and a higher left ventricular ejection fraction (LVEF) at discharge (60 (56–64) % vs. 57 (51–62)%, *P* = 0.007). The estimated glomerular filtration rate (eGFR) in the DNC group was higher when the patient was transferred to the intensive care unit (79.4 (65.0-94.3) ml/min/1.73m^2^ vs. 77.2 (59.8–88.7) ml/min/1.73m^2^, *P* = 0.014), but no significant differences were identified after 24 h. The serum lactate values of the DNC group were significantly lower than those of the CBC group (0 h: 2.7 (2.0-3.2) vs. 3.2 (2.4–4.4), *P* = 0.001; 3 h: 3.2 (2.0-4.8) vs. 4.8 (2.8–6.6), *P* < 0.001; 6 h: 3.5 (2.2–5.4) vs. 5.8 (3.4–8.4), *P* < 0.001; 9 h: 3.4 (2.0–7.0) vs. 5.5 (2.9–8.3), *P* = 0.005). There were no differences between the two groups in respect of lactate levels at 12 h and thereafter. Postoperative creatinine kinase-MB concentrations were similar between the two groups.

**Conclusions:**

Del-Nido cardioplegia is safe and effective in elderly patients undergoing CABG and/or valve surgery.

**Supplementary Information:**

The online version contains supplementary material available at 10.1186/s13019-023-02269-3.

## Introduction

Safe and reliable myocardial protection is an essential component of cardiac surgery. Since 1958, Gerbode and Melrose have used potassium citrate to induce cardiac arrest in humans [1], and significant efforts have been made to identify the optimal myocardial protection strategy. There are two types of cardioplegia: cold crystalloid cardioplegic solutions and hyperkalemic blood cardioplegia. Although cold blood cardioplegia (CBC), usually a 4:1 blood-to-crystalloid mixture, has been extensively used since the 1980s, CBC still needs to overcome the shortcomings of high total solution volume and short infusion interval.

Del-Nido cardioplegia (DNC) has been demonstrated to be safe and effective for infant and pediatric cardiac surgery since 1995 [2]. It is comprised of a base solution of plasma-Lyte, which is similar to extracellular fluid and oxygenated autologous blood in a 4:1 ratio [3, 4]. In addition to potassium chloride, mannitol, magnesium sulfate, sodium bicarbonate, and lidocaine are also required for hyperkalemic arrest, reducing myocyte edema, scavenging free radicals, and preventing intracellular calcium accumulation. The infusion interval of DNC is 60 to 90 min [4]. DNC has also been used in adult cardiac surgery, such as coronary artery bypass grafting (CABG) and valve surgeries. Existing studies reveal the effective and safe myocardial protection of DNC [5–7]. The aim of the present study was to evaluate the safety of DNC in patients over 70 years old who underwent cardiac surgery.

## Methods

The present retrospective observational study was approved by the Research Ethics Committee of Guangdong Provincial People’s Hospital, and the requirement for patient-informed consent was waived.

### Patients characteristics

From January 2018 to August 2022, 302 patients aged 70 years or older who underwent on-pump valve surgery and/or CABG were included. DNC was used in 90 patients, and CBC was used in 212 patients. The selection of cardioplegia depended on the surgeon’s preference. Patients who had previous cardiac surgery were also included. There were no exclusion criteria in the present study.

The demographic characteristics included age, gender, NYHA classification, common commodities, and EuroSCORE II result. To evaluate the left ventricular ejection fraction (LVEF), transthoracic echocardiography (TTE) was performed preoperatively and before discharge.

### Myocardial protection technique

CBC was a 4:1 mixture of oxygenated autologous blood from the cardiopulmonary bypass (CPB) circuit and crystalloid (**Supplement Table**). The initial dosage was 20ml/kg, with a maximum volume of 1000ml. An additional 10ml/kg (up to 500ml) of CBC was administered every 30 min during the aortic cross-clamp period; the potassium concentration of the subsequent doses was half of the first dose.

DNC has been used since January 2018. The oxygenated autologous blood was combined with the prepared crystalloid in a 1:4 ratio (**Supplement Table**). The first dosage was 20ml/kg (maximum volume: 1000ml), and a subsequent 10ml/kg (maximum volume: 500 ml) of DNC was given every 60 min or when spontaneous electrical occurred.

For the two groups, the target pressure of infusion was 100 to 200 mmHg, and the target infusion flow was 200 to 300 ml/min. The cardioplegia was delivered anterogradely through aortic root cannulation or by direct ostial delivery. Retrograde delivery was not routinely used unless there was severe stenosis of the coronary arteries or requested by the surgeon. Topical hypothermia via ice slush and systematic hypothermia to 30℃ were used in all the patients.

### Post-operative outcomes

Post-operative outcomes were evaluated during the corresponding hospitalization. Primary outcomes included death, intra-aortic balloon pump (IABP) implantation, and extracorporeal membrane oxygenation (ECMO) implantation. Secondary outcomes included post-operative LVEF (using Simpson’s method, TTE), level of inotropes index (defined as dopamine dose(µg/kg·min) + dobutamine dose (µg/kg·min) + 10×milrinone dose (µg/kg·min) + 100×epinephrine dose (µg/kg·min) + 100×noradrenaline dose(µg/kg·min)), creatine kinase-MB (CK-MB) and estimated glomerular filtration rate (eGFR, patients who underwent dialysis for end-stage kidney failure were excluded in the post-operative analysis) at 4-time points: baseline, admission to the intensive care unit (ICU), 24 and 48 h after admission to the ICU, acute kidney injury (KIDGO criteria, serum creatinine increased ≥ 0.3 mg/dl within 48 h or ≥ 1.5-fold from baseline), postoperative hospital length of stay, ICU length of stay, ventilation duration, blood product transfusion (any or more of the following: packed red blood cell, platelet, fresh frozen plasma), dialysis, stroke, reoperation, and surgical-site infection. The lactate level was obtained from arterial blood gas analysis every three hours since admission to the ICU.

### Statistical analyses

All statistical analyses were conducted using IBM SPSS Statistics for macOS, Version 25.0 (Released 2017, IBM Corporation, Armonk, NY, USA), and graphics were designed with GraphPad Prism, Version 9.00 for macOS (GraphPad Software, La Jolla, CA, USA) and RStudio Team (2020) (RStudio: Integrated Development for R. RStudio, PBC, Boston, MA URL).

The continuity variables are presented as median (25th to 75th percentile). Categorical data are presented as a number (frequency, %). The differences between the DNC group and the CBC group were analyzed by Mann-Whitney U-test for continuous variables. χ^2^ or Fisher exact tests for categorical variables and odds ratio was calculated in the post-operative outcomes. The statistical significance was set as *P* < 0.05.

The baseline characteristics were compared between the DNC group and the CBC group. Propensity score matching was performed to adjust the significantly different baseline characteristics. To calculate the propensity score for each patient, a logistic regression model was adopted with a 1:1 nearest-neighbor matching algorithm and a caliper of 0.1 [8]; said model included the following covariates: age, gender, NYHA III/IV, body mass index, EuroSCORE II, pre-operative eGFR, LVEF, atrial fibrillation, hypertension, diabetes, coronary artery disease (CAD), history of percutaneous coronary intervention (PCI), active infectious endocarditis (IE), chronic obstructive pulmonary disease (COPD), dialysis, peripheral artery disease (PAD), previous cardiac surgery, pre-operative ventilator support and use of inotrope. The standardized mean difference (SMD) was calculated to assess the balance of matching [9].

## Results

The present study consisted of 90 patients in the DNC group and 212 patients in the CBC group. All the patients received anterograde cardioplegia delivery. The overall median age was 72 (Range: 70–89, 25th -75th quartile: 72–74) years. There were 168 (55.6%) male patients. The general EuroSCORE II of the whole cohort was 1.9 (Range: 0.8–44.4, 25th -75th quartile: 1.4–3.3). Based on the results of univariate analysis between the unmatched groups, there were significant differences in the baseline characteristics. Compared with the CBC group, those in the DNC group had a lower incidence of hypertension (*P* = 0.039) but a higher incidence of COPD (*P* = 0.029). After propensity score matching on twenty baseline covariates, 89 pairs of matched patients were obtained. Aside from the absolute value of the standardized mean difference (|SMD|) of eGFR being 17.0%, the |SMD| of the other nineteen baseline covariates were ≤ 10.6%, indicating that the imbalance of baseline characteristics was adjusted adequately (Table 1).

**Table 1:** Baseline characteristics before and after propensity score matching

Variable	Before propensity score matching				After propensity score matching			
	DNC (n = 90)	CBC (n = 212)	*P*	| SMD | (%)	DNC (n = 89)	CBC (n = 89)	*P*	| SMD | (%)
Age (years)	72 (71–74)	72 (70–74)	0.499	8.2	72 (71–74)	72 (70–74)	0.828	5.0
EuroSCORE II (%)	1.9 (1.4–3.2)	2.0 (1.5–3.3)	0.706	9.7	1.8 (1.4–3.1)	2.0 (1.4–3.4)	0.686	5.7
BMI (kg/m^2^)	22.1 (20.4–24.1)	22.5 (19.9–24.4)	0.770	0.4	22.1 (20.4–24.0)	22.6 (20.5–24.6)	0.413	7.4
Male (%)	49 (54.4)	119 (56.1)	0.787	3.4	49 (55.1)	53 (59.6)	0.544	9.0
NYHA III/IV (%)	69 (76.7)	168 (79.2)	0.618	6.1	68 (76.3)	70 (78.6)	0.719	5.3
eGFR (ml/min/1.73m^2^)	78.5 (64.3–94.1)	76.0 (58.8–86.7)	0.378	17.8	78.9 (64.9–94.1)	77.2 (60.2–88.4)	0.273	17.0
LVEF (%)	64 (60–69)	63 (58–67)	0.841	12.2	64 (60–69)	64 (59–66)	0.514	9.0
Comorbidities								
Af	37 (41.1)	91 (42.9)	0.771	3.7	37 (41.6)	35 (39.3)	0.760	4.5
Hypertension	35 (38.9)	110 (51.9)	***0.039***	26.5	35 (39.3)	35 (39.3)	1.000	< 0.1
Diabetes mellitus	12 (13.3)	33 (15.6)	0.618	6.5	12 (13.5)	10 (11.2)	0.649	6.6
Stroke	6 (6.7)	17 (8.0)	0.685	5.4	6 (6.7)	5 (5.6)	0.756	4.5
CAD	26 (28.9)	51 (24.1)	0.378	10.6	26 (29.2)	22 (24.7)	0.499	9.8
PCI history	7 (7.8)	8 (3.8)	0.155	14.9	7 (7.9)	5 (5.6)	0.550	8.3
COPD	16 (17.8)	19 (9.0)	***0.029***	22.9	15 (16.9)	15 (16.9)	1.000	< 0.1
Active IE	6 (6.7)	9 (4.2)	0.392	9.7	6 (6.7)	4 (4.5)	0.515	8.9
PAD	4 (4.4)	7 (3.3)	0.738	5.5	3 (3.4)	3 (3.4)	1.000	< 0.1
CKD								
stage 1–2	70 (77.8)	154 (73.0)	0.182	8.3	70 (78.7)	67 (75.3)	0.481	5.2
stage 3–4	19 (21.1)	57 (27.0)			18 (20.2)	22 (24.7)		
stage 5	1 (1.1)	0 (0.0)			1 (1.1)	0 (0.0)		
Previous cardiac surgery	4 (4.4)	6 (2.8)	0.492	7.8	4 (4.5)	3 (3.4)	1.000	5.4
Preoperative ventilator	1 (1.1)	1 (0.5)	0.508	6.1	1(1.1)	0 (0.0)	1.000	10.6
Preoperative inotropes	1 (1.1)	2 (0.9)	1.000	1.6	1 (1.1)	1 (1.1)	1.000	< 0.1

Values are presented as median (25th quartile, 75th quartile) or n (%). The differences between the two groups were analyzed by Mann-Whitney U-test for continuous variables and χ^2^ or Fisher exact tests for categorical variables. The statistical significance was set as *P* < 0.05. DNC, Del-Nido cardioplegia; CBC, cold blood cardioplegia; SMD: standardized mean difference; BMI: body mass index; eGFR: estimated glomerular filtration rate; LVEF, left ventricular ejection fraction; Af, atrial fibrillation; CAD, coronary artery disease; PCI, percutaneous coronary intervention; COPD, chronic obstructive pulmonary disease; IE, infectious endocarditis; PAD, peripheral artery disease; CKD, chronic kidney disease

### Intra-operative result

The DNC group and the CBC group were similar in respect of CPB time, aortic cross-clamp time, nadir bladder temperature, hematocrit during CPB, and oxygen delivery index (DO_2_i) during CPB. Compared with the CBC group, the DNC group had a small volume of infused cardioplegia (*P* < 0.001) and fewer infusion times (*P* < 0.001). There were no significant differences in the distribution of the concomitant cardiac surgeries between the two groups, including mitral valve repair, mitral valve replacement, aortic valve replacement, tricuspid valve repair, CABG, and Maze procedure. The rate of video-assisted thoracoscopic cardiac surgery was similar between the two groups (Table 2).

**Table 2:** Perioperative characteristics of matched patients

Variable	DNC (n = 89)	CBC (n = 89)	*P* value
CPB time (min)	133 (106–169)	133 (108.5-171.5)	0.607
Aortic cross clamp time (min)	86 (65.5–105)	83 (65-110.5)	0.816
Infused cardioplegia volume (ml)	1500 (1350–1500)	2000 (2000–2500)	***< 0.001***
Cardioplegia infusion times			
1	16 (18.0)	1 (1.1)	***< 0.001***
2	62 (69.7)	16 (18.0)	
3	11 (12.3)	39 (43.8)	
≥4	0 (0.0)	33 (37.1)	
Nadir bladder temperature (℃)	31.5 (30.6–32.4)	30.4 (29.8–31.1)	***< 0.001***
Nadir hematocrit during CPB (%)	25 (23–26)	25 (23–26)	0.672
Nadir DO_2_i during CPB	331.3 (312.2-355.1)	323.7 (308.4-358.2)	0.405
Concomitant cardiac surgeries			
Mitral valve repair	15 (16.8)	23 (25.4)	0.143
Mitral valve replacement	48 (53.9)	41 (46.1)	0.294
Aortic valve replacement	31 (34.8)	38 (42.7)	0.282
Tricuspid valve repair	35 (39.3)	40 (44.9)	0.448
CABG	11 (12.4)	15 (16.9)	0.396
Maze procedure	6 (6.7)	6 (6.7)	1.000
VATS surgeries	10 (11.2)	11 (12.4)	0.816
Postoperative hospital stay (days)	11 (8-16.5)	13 (7–20)	0.343
ICU stay (hours)	64 (41.5-114.5)	70 (45-171.5)	0.108
Ventilation duration (hours)	24 (15.5–54)	23 (16–62)	0.777
Inotrope index			
0 h post-op.	5 (4–10)	6 (2.8–12)	0.947
24 h post-op.	3 (1-7.8)	3.5 (0–10)	0.898
48 h post-op.	0 (0–5)	0 (0–5)	0.988
LVEF at discharge (%)	60 (56–64)	57 (50.5–62)	***0.007***

Values are presented as median (25th quartile, 75th quartile) or n (%). DNC, Del-Nido cardioplegia; CBC, cold blood cardioplegia; CPB, cardiopulmonary bypass; DO_2_i, oxygen delivery index; CABG, coronary artery bypass grafting; VATS, video-assisted thoracoscopic surgery; ICU, intensive care unit; LVEF, left ventricular ejection fraction

### Post-operative outcomes

**Fig. 1:**
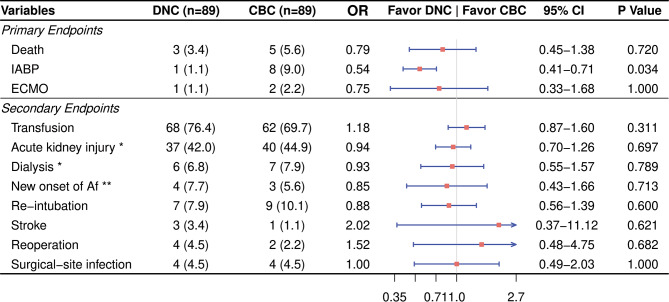
Outcomes of propensity score-matched patients. Values are presented as n (%). DNC, Del-Nido cardioplegia; CBC, cold blood cardioplegia; OR, odds ratio; CI, confident interval; IABP, intra-aortic balloon pump; ECMO, extracorporeal membrane oxygenation; Af, atrial fibrillation. *Patient with CKD-5 was excluded from the DNC group (88 cases remaining). **Calculated on patients without Af pre-operatively, 4 of 52 in the DNC group and 3 of 54 in the CBC group

The DNC group had a significantly lower rate of IABP implantation (1.1% vs. 9.0%, OR = 0.54, 95% CI 0.41–0.71, *P* = 0.034). And the mortality and the rate of ECMO implantation were similar between the two groups. There were no differences in the transfusion rate, length of ICU stay, length of hospital stay, ventilation duration, post-op inotrope index, and complications such as the new onset of atrial fibrillation, re-intubation, acute kidney injury, dialysis, stroke, reoperation, and surgical-site infection (Fig. 1).

### Change of LVEF and CK-MB

Compared with the pre-operative LVEF, the LVEF at discharge decreased in both groups (the DNC group: 60 (56–64) % vs. 64 (60–69) %, *P* < 0.001; the CBC group: 57 (50.5–62) % vs. 64 (59–66) %, *P* < 0.001). Although the levels of pre-operative LVEF were the same in both groups, the patients had higher LVEF at discharge in the DNC group (60 (56–64) vs. 57 (50.5–62), *P* = 0.007).

**Fig. 2:**
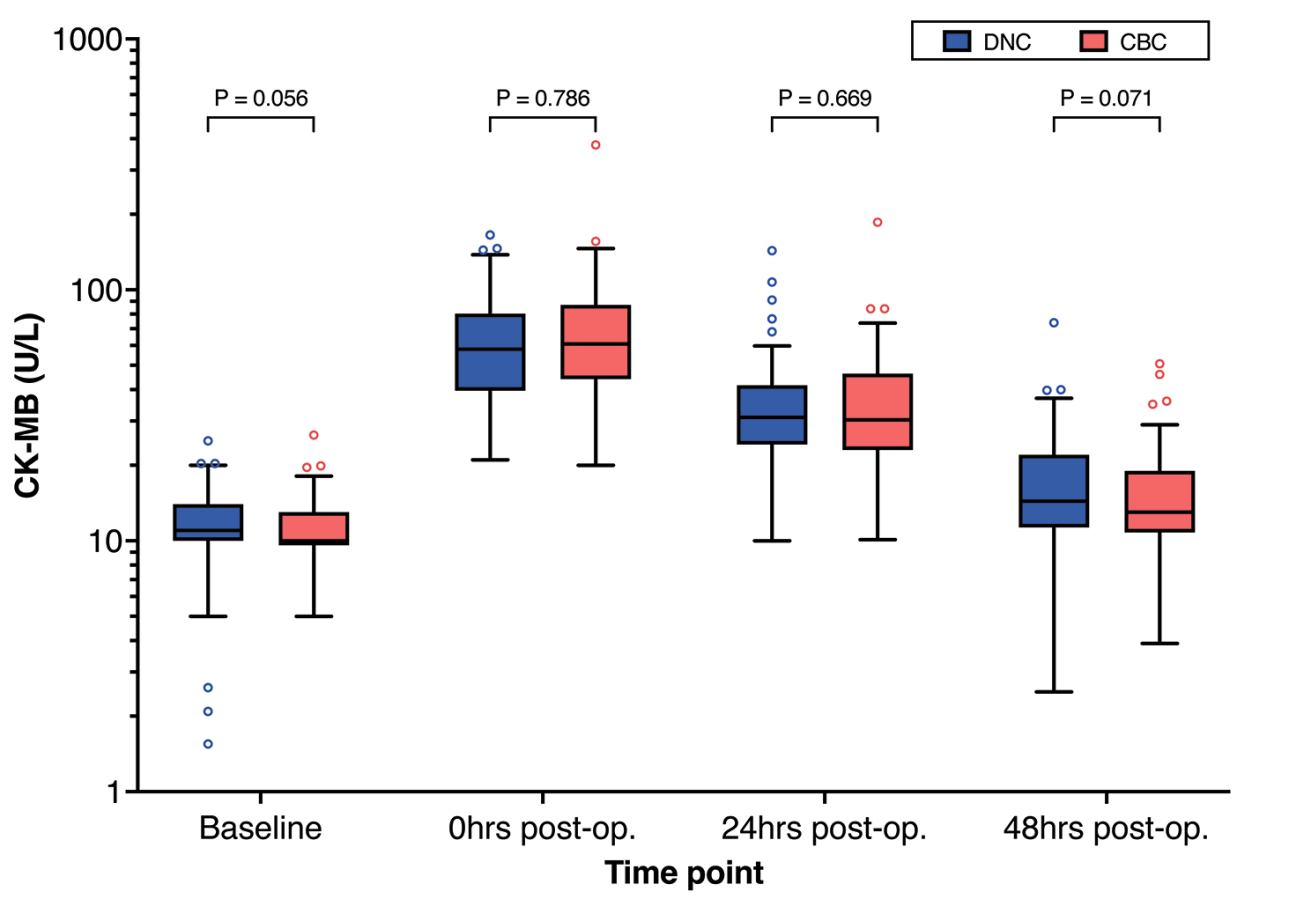
Matched comparison of serum CK-MB level between the DNC group and the CBC group. Values were compared at four time points: preoperative baseline, 0 h, 24 h, and 48 h post-operation

The serum CK-MB levels between the two groups were similar at baseline and 0 h, 24 h, and 48 h post-operatively (Fig. 2).

### Change of estimated glomerular filtration rate

**Fig. 3:**
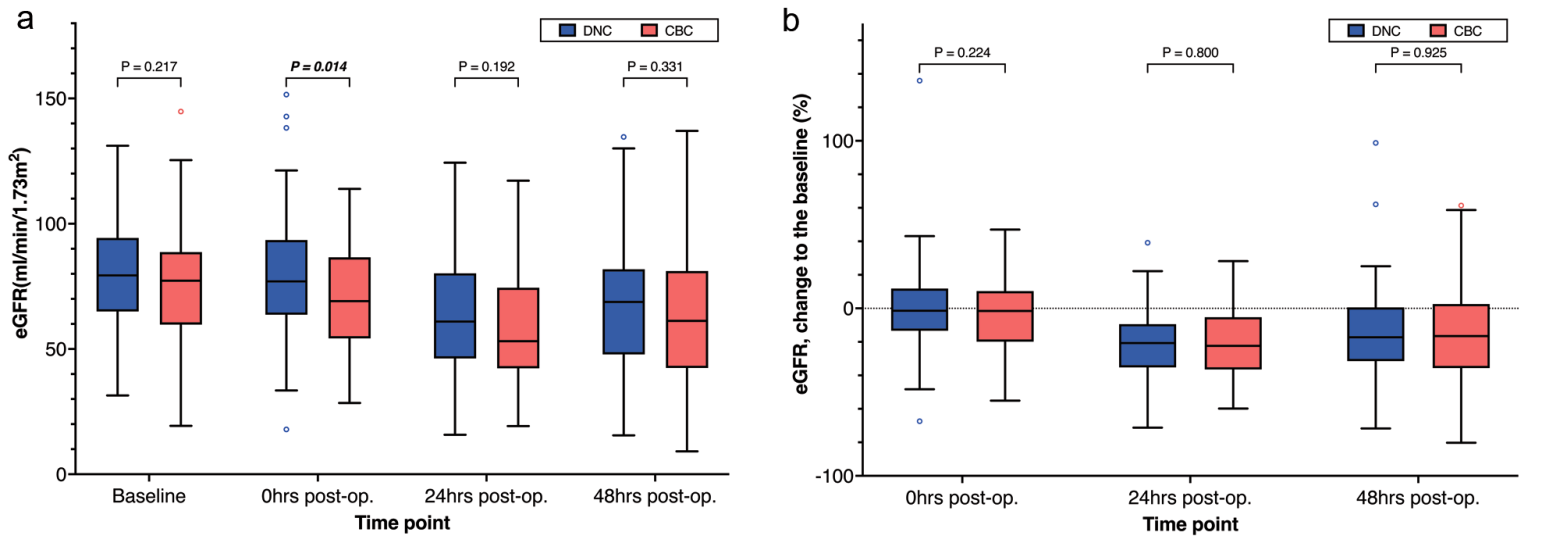
Matched comparison of values(3 A) and change rate(3B) to baseline of estimated glomerular filtration rate between the DNC group and the CBC group. Values were compared at four time points: preoperative baseline, 0 h (*P* = 0.021), 24 h, and 48 h post-operation

For patients without pre-operative dialysis, the eGFR in the DNC group was higher when the patient was transferred to ICU, than that in the CBC group (*P* = 0.014), despite the baseline eGFR of the two groups being similar. At 24 and 48 h, the eGFR did not differ significantly between the two groups. The eGFR change rate to baseline showed no differences at 0,24 and 48 h. (FIGURE 3).

### Change in serum lactate levels

**Fig. 4:**
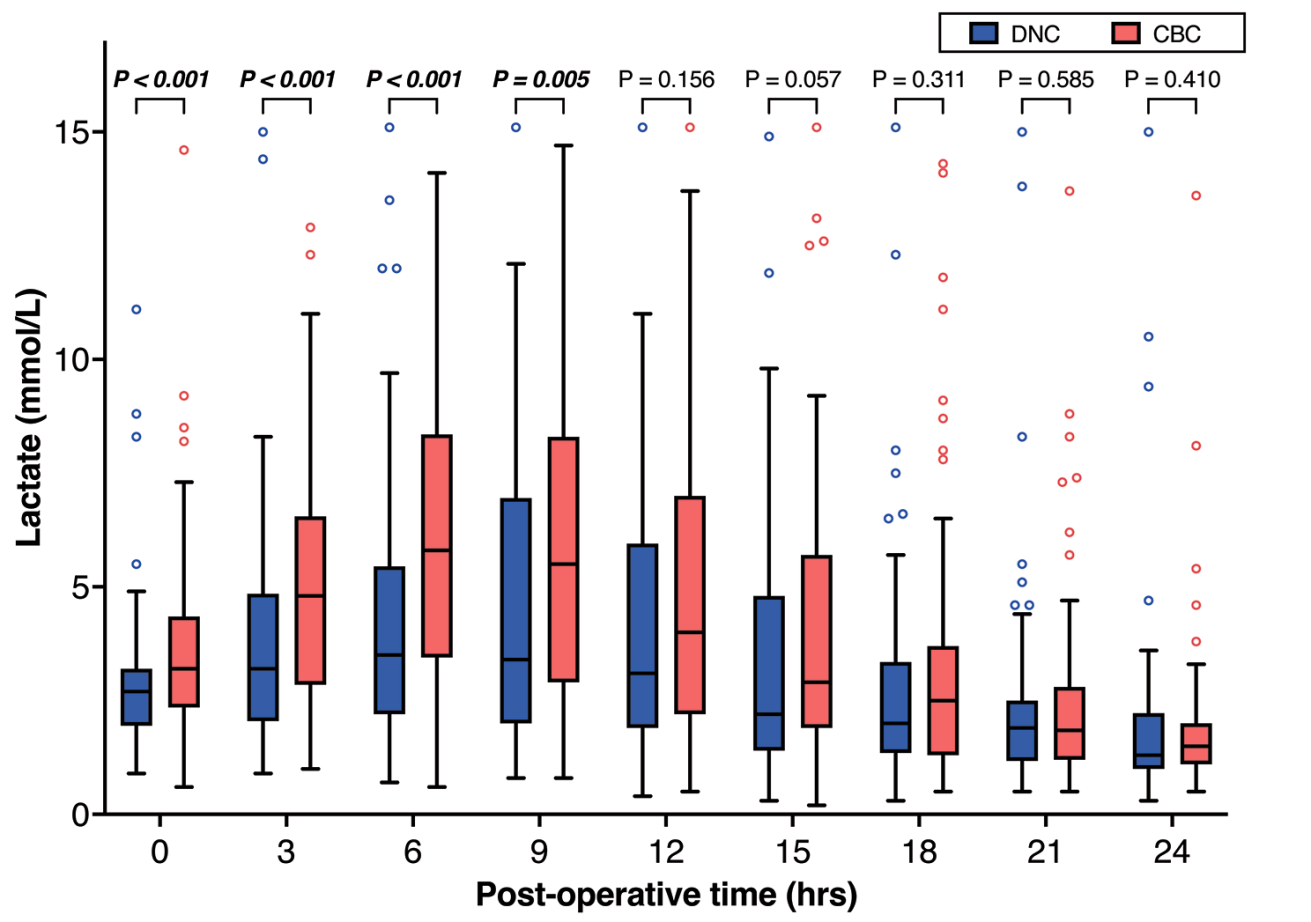
Matched comparison of post-operative lactate level in arterial blood between the DNC group and the CBC group

The serum lactate level was analyzed every three hours after surgery. From the time the surgery was completed to nine hours after surgery, the lactate levels of the DNC group were significantly lower than those of the CBC group in the early stage. There were no differences between the two groups in respect of lactate levels at 12 h and thereafter (Fig. 4).

## Discussion

For elderly patients requiring cardiac surgery, optimal myocardial protection strategies remain controversial, especially the choice of cardioplegia, with there being a scarcity of research thereon. In the present study, the suggestion is that DNC is safe and effective in valve and/or CABG surgeries for patients over 70 years old. Compared with traditional cold blood cardioplegia, DNC is associated with a lower incidence of IABP implantation, higher postoperative LVEF, lower early lactate levels, and preserved renal function.

After myocardial damage occurs, CK-MB increases within the first hour and reaches peak levels between 16 and 30 h before finally returning to normal by 24 to 36 h. A recent randomized prospective study by Sanetra et al. suggested that CK-MB and troponin values at 24 and 48 h were similar between DNC and CBC groups, the patients of which underwent aortic valve replacement and had a mean age of 63 years old [10]. Another prospective study conducted by Niv Ad et al. compared the postoperative troponin I level in patients with a mean age of 65 years old requiring first-time CABG and/or valve surgeries. For the DNC group, the troponin levels thereof did not increase as much as those of the whole blood cardioplegia group (*P* = 0.040) [7]. In other retrograde studies, DNC was associated with a lower or similar level of CK-MB or troponin in patients who underwent CABG, valvular or aortic surgeries [5, 11–13]. In this study, DNC is associated with a lower rate of IABP while maintaining similar post-operative CK-MB levels compared with CBC, indicating that DNC was less likely to cause myocardial stunning, which might be an underlying cause for low cardiac output syndrome.

In the present study, the postoperative ejection fraction decreased in all patients compared with the baseline, which is quite common. However, compared with the CBC group, the postoperative LVEF was significantly preserved in the DNC group (60 (56–64) vs. 57 (50.5–62), *P* = 0.007). Lenoir et al. found no difference in the LVEF on postoperative echocardiography between DNC and CBC groups in a propensity score-matched analysis of 283 consecutive aortic root surgery cases, although the LVEF decreased in both groups compared to the pre-operative baseline [14]. Similar changes in postoperative LVEF were also observed in valve surgery, aortic dissection surgery, and minimally invasive aortic valve surgery [3, 6, 11, 14]. In another propensity score-matched analysis by Timek et al., 851 consecutive isolated CABG cases were analyzed. Since there were no differences in postoperative LVEF between DNC and CBC groups, a subgroup analysis of patients with pre-operative LVEF ≤ 35% revealed an increasing trend of LVEF after surgery in both groups (pre-operative LVEF, CBC group: 26 ± 7, DNC group: 30 ± 6, *P* = 0.06; post-operative LVEF, CBC group: 41 ± 14, DNC group: 43 ± 12, *P* = 0.58), which could be explained by satisfactory revascularization [13]. [3, 6, 11, 14]

Serum lactate concentration represents the perfusion of critical organs, and a high serum lactate level after cardiac surgery is associated with the low cardiac output after excluding hypovolemia [15, 16]. In the center of the present authors, serum lactate is one of the most frequently used biomarkers to monitor cardiac output and vital organ perfusion due to serum lactate being quickly obtainable from arterial blood gas analysis. In the present study, the lactate levels of the DNC group were significantly lower than the CBC group in the first 9 h after surgery. After reaching the peak at about 9 h, the lactate concentration decreased in both groups, and no differences were identified after 12 h. Hyperlactatemia is raised from accelerated anaerobic metabolism as a consequence of impaired tissue perfusion [17]. At the same time, the DNC group in the present study had a low incidence of IABP implantation, indicating a better early-stage effect of myocardial protection or preservation of cardiac output and peripheral perfusion.

As a major complication of on-pump cardiac surgeries, acute kidney injury (AKI) due to ischemia-reperfusion injury is associated with increased mortality and morbidity [18]. Age is an independent risk factor for AKI related to cardiac surgery, especially in patients older than 70 years (RR:1.79, 95% CI:1.17–2.72) [19]. In the present study focusing on patients over 70 years, the eGFR of the DNC group was higher than the CBC group immediately after surgery. Although there was no significant difference in the baseline eGFR between the two groups, the eGFR continued to decrease in all of the patients until reaching 48 h, but no significance in eGFR was observed after 24 h. Similar results in younger patients have been reported. Sanetra et al. reported that patients who underwent aortic valve replacement and adopted DNC had a relatively low incidence of kidney injury according to the classification of the Acute Kidney Injury Network (AKIN), but the mean serum creatinine levels during hospitalization were similar between the DNC and CBC groups [10]. In further CABG and/or other valve surgery studies, non-inferior results of renal impairment were reported in DNC groups compared with other cardioplegias [7, 12]. However, it was proved that insufficient oxygen delivery was associated with an increased incidence of AKI [20]. In our study, there were no differences in nadir hematocrit and oxygen delivery index between the two groups, which suggested kidney perfusion during CPB was similar. However, a higher eGFR was detected in the DNC group immediately after surgery. One possible explanation could be that the early-stage cardiac output, especially after weaning from CPB, was higher in the DNC group due to better myocardial protection. Further study is warranted at several levels to increase our understanding of the early-stage effect of DNC.

## Limitations

There were several limitations in the present study. The present study involved a single-center retrograde analysis, and inherent selection bias could not be ignored. The cardioplegia was determined by the surgeon, and there was no randomization or blinding. Thus, the bias of a certain surgeon in favor of certain cardioplegia would be inevitable. The sample size was relatively small.

## Conclusion

In the present study, DNC is a safe and efficient cardioplegia for elderly patients. There were no differences in the postoperative CK-MB levels between DNC and CBC. Compared with CBC, DNC was associated with a lower incidence of IABP implantation, lower early serum lactate concentration, higher early eGFR, and preserved LVEF.

## Electronic supplementary material

Below is the link to the electronic supplementary material.

Additional File: Formula of cardioplegia

## Data Availability

The datasets used and/or analyzed during the current study are available from the corresponding author upon reasonable request.

## Abbreviations

DNC: Del-Nido cardioplegia
CBC: Cold blood cardioplegia
CABG: Coronary artery bypass graft
IABP: Intra-aortic balloon pump
LVEF: Left ventricular ejection fraction
eGFR: Estimated glomerular filtration rate
CK-MB: Creatinine kinase-MB
TTE: Transthoracic echocardiography
CPB: Cardiopulmonary bypass
ECMO: Extracorporeal membrane oxygenation
ICU: Intensive care unit
CAD: Coronary artery disease
PCI: Percutaneous coronary intervention
IE: Infectious endocarditis
COPD: Chronic obstructive pulmonary disease
PAD: Peripheral artery disease
SMD: Standardized mean difference
DO_2_i: Oxygen delivery index
AKI: Acute kidney injury
Af: Atrial fibrillation
