# Supplementary material for: Del-Nido cardioplegia in cardiac surgery for elderly patients: a propensity score-matched analysis
Source: J Cardiothorac Surg. 2023 Apr 25;18:161. doi: 10.1186/s13019-023-02269-3 (PMC10131453; doi:10.1186/s13019-023-02269-3)
Supplement: Supplementary file 1 — Additional File: Formula of cardioplegia [file 13019_2023_2269_MOESM1_ESM.pdf]

Supplement Table. Formula of cardioplegia

	DNC	CBC	
		Initial dosage	Sequential dosage
Volume	Initial: 20ml/kg, up to 1000ml<br>Sequential: 10ml/kg, up to 500ml	20ml/kg, up to 1000ml	10ml/kg, up to 500ml
Composition (per set)			
Base solution	Plasma-Lyte A 500ml	NS 500ml	NS 500ml
25% $MgSO_4$	4ml	19.2ml	19.2ml
10% KCl	10ml	35ml	14ml
2% Lidocaine	3.25 ml	10ml	2.5ml
20% mannitol	8.15ml		
5 % $NaHCO_3$	11ml		
Final concentration of ions			
K^+	24 mEq/L	20 mEq/L	10 mEq/L
Mg^{2+}	12 mEq/L	14 mEq/L	14 mEq/L
